# Revealing autoimmune gastritis: Polypoid nodule scar development after endoscopic submucosal dissection for early gastric cancer

**DOI:** 10.1002/deo2.70094

**Published:** 2025-03-11

**Authors:** Naoya Masuda, Kenji Yamazaki, Yasuhiko Maruyama, Ryoji Kushima, Nae Hasebe, Noritaka Ozawa, Shogo Shimizu, Masahito Shimizu

**Affiliations:** ^1^ Department of Gastroenterology Gifu Prefectural General Medical Center Gifu Japan; ^2^ Department of Gastroenterology Gifu University School of Medicine Gifu Japan; ^3^ Department of Gastroenterology Fujieda Municipal General Hospital Shizuoka Japan; ^4^ Department of Pathology Shiga University of Medical Science Shiga Japan

**Keywords:** autoimmune gastritis, endoscopic submucosal dissection, gastrin, *Helicobacter pylori*, polypoid nodule scar

## Abstract

Endoscopic submucosal dissection (ESD), the standard treatment for early gastric cancer, typically results in homogeneous flat scars. However, in some cases, polypoid nodule scars (PNS) may develop, complicating the cancer recurrence assessment. This case report describes a 60‐year‐old man with a history of *Helicobacter pylori* infection who underwent two ESD procedures: first for early antral gastric cancer and then for gastric body adenoma. Subsequently, an erythematous protruding lesion developed at the antral ESD scar site. Lesion biopsy revealed regenerative and hyperplastic tissue growth, consistent with PNS. Despite *H. pylori* eradication therapy and discontinuation of potassium‐competitive acid blockers and H_2_‐receptor antagonists, the lesion continued to enlarge. PNS growth may be caused by excessive mucosal regeneration and enhanced antral peristalsis, suggesting that hypergastrinemia, which may enhance these effects, may be an underlying cause. Further, elevated serum gastrin levels, decreased pepsinogen levels, the presence of antiparietal cell antibodies, and consistent pathological findings confirmed autoimmune gastritis (AIG).

This case highlights the diagnostic challenges of AIG, especially in cases of active or previous *H. pylori* infection because typical endoscopic features may be obscured. Persistent PNS after ESD warrants the consideration of excessive mucosal regeneration and enhanced peristalsis, with AIG as a potential cause because of its association with hypergastrinemia. To our knowledge, this is the first case report describing a potential link between AIG and PNS.

## INTRODUCTION

Endoscopic submucosal dissection (ESD) is the preferred treatment for early gastric cancer with no or low risk of lymph node metastasis,^1^ after which postoperative scars typically appear homogeneous and whitish, often with converging folds. Arantes et al. reported that post‐ESD endoscopy can reveal irregular scarring and large protruding nodules, particularly in the gastric antrum, termed polypoid nodule scars (PNS),^2^ which can complicate the evaluation of post‐ESD cancer recurrence.

Autoimmune gastritis (AIG) is characterized by hypergastrinemia and the autoimmune destruction of parietal cells, leading to the production of autoantibodies including antiparietal cell antibodies (APCA), against proton pumps (H+/K+ ATPase). Various triggers for AIG diagnosis have been reported, including endoscopic screening, macrocytic anemia, iron deficiency anemia, thyroid disease, gastric cancer, and close examination of gastric neuroendocrine tumors.^3^, ^4^, ^5^ Despite new Japan Gastroenterological Endoscopy Society criteria,^6^ diagnosing AIG is challenging with active or previous *Helicobacter pylori* infection, and efforts are ongoing to establish reliable diagnostic methods for such cases.

In this report, we describe the case of a 60‐year‐old man with a history of *H. pylori* infection who underwent ESD and subsequently developed persistent PNS. Despite the lack of typical endoscopic features of AIG, the progressive nature of PNS led us to consider excessive mucosal regeneration and enhanced antral peristaltic activity, suggesting hypergastrinemia as a potential cause, ultimately leading to a diagnosis of AIG.

## CASE REPORT

A 60‐year‐old man with a history of hypertension, type 2 diabetes, and mild gastroesophageal reflux disease was referred to our hospital for further evaluation after undergoing an upper gastrointestinal endoscopy during a routine physical examination. Endoscopy suggested *H. pylori* infection and early cancer of the gastric antrum.

**FIGURE 1:**
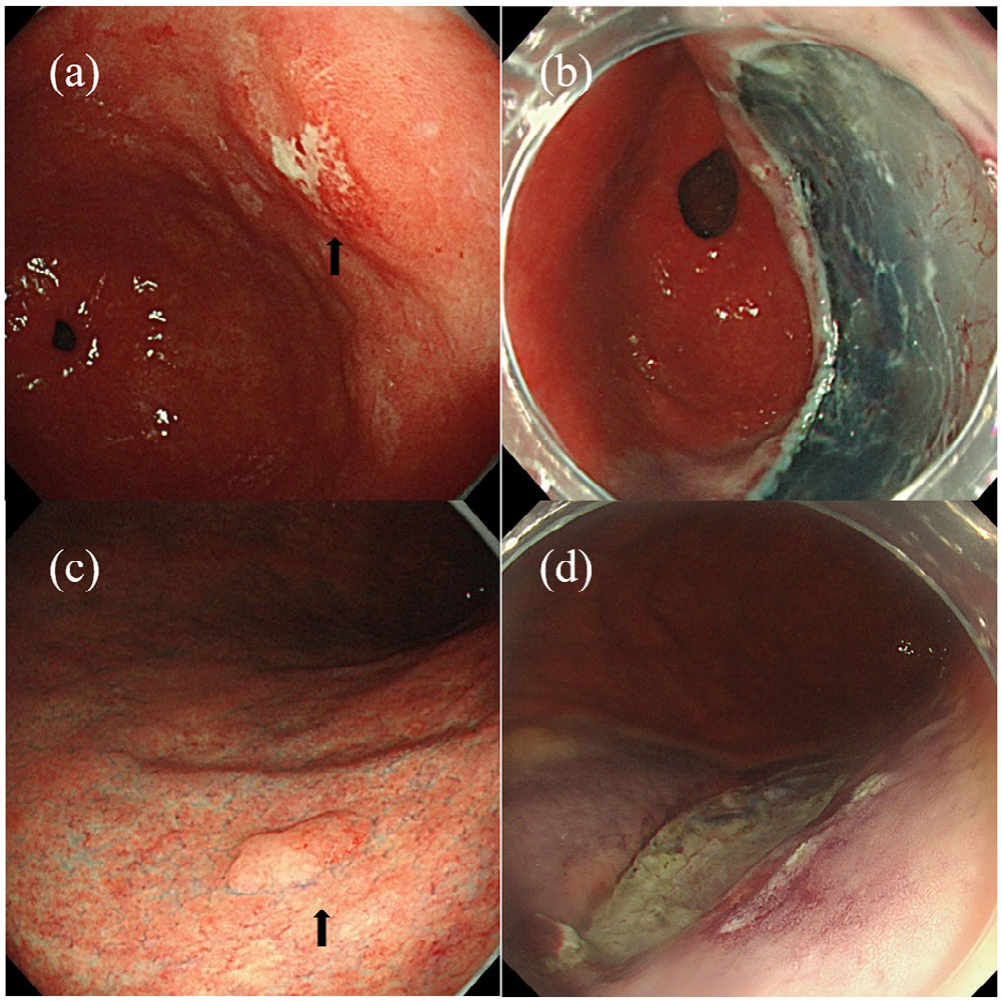
(a) Type 0–IIc early gastric cancer is approximately 10 mm in diameter, erythematous, and located on the posterior wall of the gastric antrum. The background mucosa shows atrophic changes and intestinal metaplasia. (b) The resection site after endoscopic submucosal dissection. (c) An intestinal adenoma, approximately 5 mm in diameter, is observed on the posterior wall of the gastric body. Background mucosal atrophy is also observed. (d) The resection site after endoscopic submucosal dissection.

After a thorough endoscopic examination at our hospital, early gastric cancer in the posterior wall of the gastric antrum was confirmed. ESD was performed to remove cancerous lesions (Figure 1a,b). The resected specimen was pathologically diagnosed as a well‐differentiated adenocarcinoma with a resection diameter of 35 × 28 mm, negative margins, and intramucosal depth. ORISE ProKnife 2.0 (Boston Scientific Co.) and a hyaluronic acid solution were used for local injection. The patient was treated postoperatively with 20 mg of vonoprazan, a potassium‐competitive acid blocker (P‐CAB), and recovered uneventfully.

Five months later, a second ESD was performed to remove the adenoma found on the posterior wall of the gastric body during the initial endoscopy (Figure 1c,d), which was pathologically diagnosed as an intestinal‐type adenoma measuring 25 × 25 mm with negative margins. The same device and local injection technique were used, and the patient again received postoperative vonoprazan with a favorable recovery. After endoscopic treatment, *H. pylori* eradication therapy was performed. Successful eradication was confirmed by a urea breath test 10 months after the first ESD.

**FIGURE 2:**
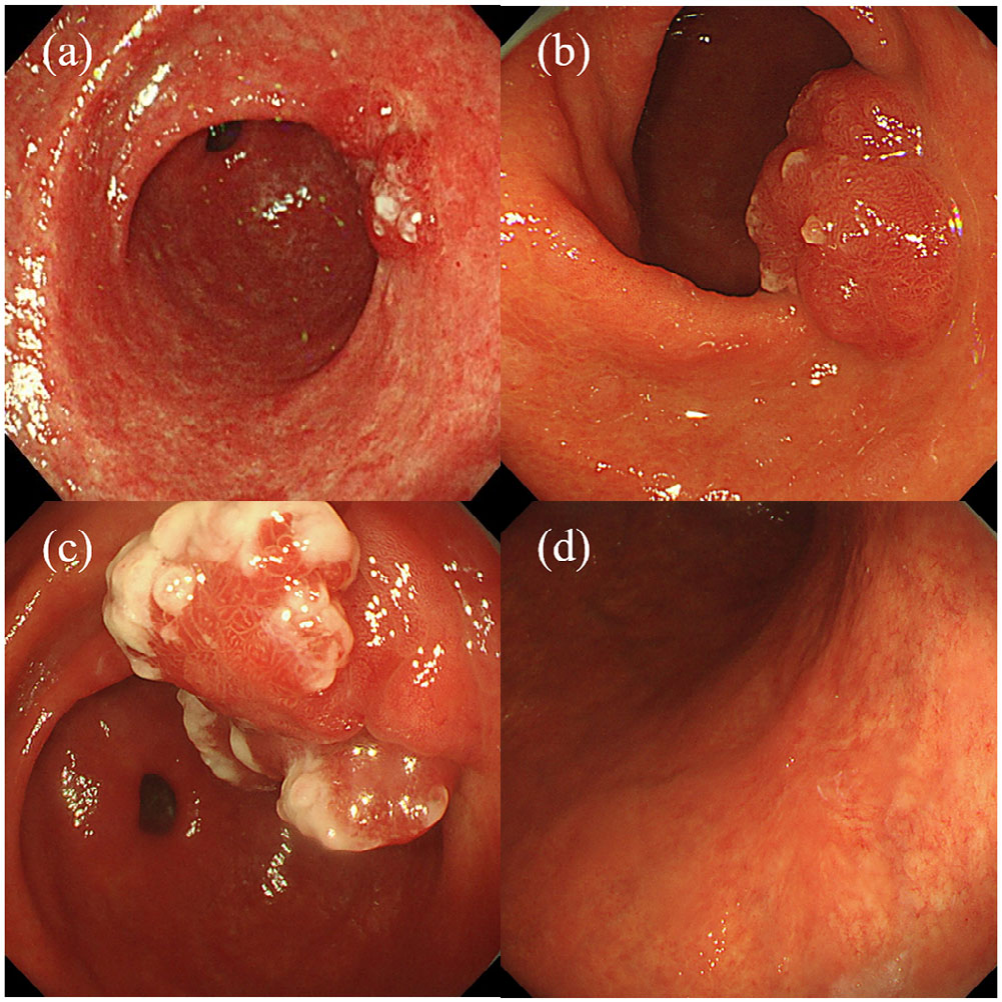
(a) Two months after the first endoscopic submucosal dissection (ESD) of the gastric antrum, the polypoid nodule scar (PNS) has gradually enlarged to approximately 20 mm in diameter. (b) Five months after the first ESD of the gastric antrum, the PNS has continued to enlarge, reaching approximately 30 mm in diameter and exhibiting an increasingly irregular shape, despite switching from vonoprazan to H_2_‐receptor antagonist therapy. (c) Ten months after the first ESD of the gastric antrum, the PNS has continued to enlarge, reaching approximately 35 mm in diameter with a rough lobulated surface, despite successful *H. pylori* eradication. (d) Two years after the second ESD of the gastric body, no PNS is observed in the post‐ESD ulcer area of the gastric body.

Two months after the first ESD of the gastric antrum, a gradually enlarging, erythematous, and irregularly protruding lesion with white moss‐like adhesions emerged at the scar site (Figure 2a). Lesion biopsy revealed regenerative and hyperplastic tissue without local cancer recurrence, consistent with PNS. Suspecting a possible adverse effect of P‐CAB, prescribed for the treatment of post‐ESD ulcers, we unavoidably switched medication to H_2_‐receptor antagonists (H2RA) for mild heartburn. However, the lesion continued to grow (Figure 2b). Despite successful *H. pylori* eradication, the protruding lesion in the gastric antrum persisted and enlarged (Figure 2c). However, the PNS was not observed in the post‐ESD gastric body ulcer area (Figure 2d).

Excessive mucosal regeneration and increased peristaltic antral activity have been reported as potential causes of PNS.^7^ In this case, despite the eradication of *H. pylori* and adjustments to medications, potentially contributing to excessive mucosal regeneration,^2^, ^7^ the PNS continued to enlarge. Hypergastrinemia has also been implicated as a contributing factor in these processes.^8^, ^9^ Therefore, biopsies of the gastric antrum and body and serum tests were conducted to evaluate AIG as a differential cause of hypergastrinemia.

**FIGURE 3:**
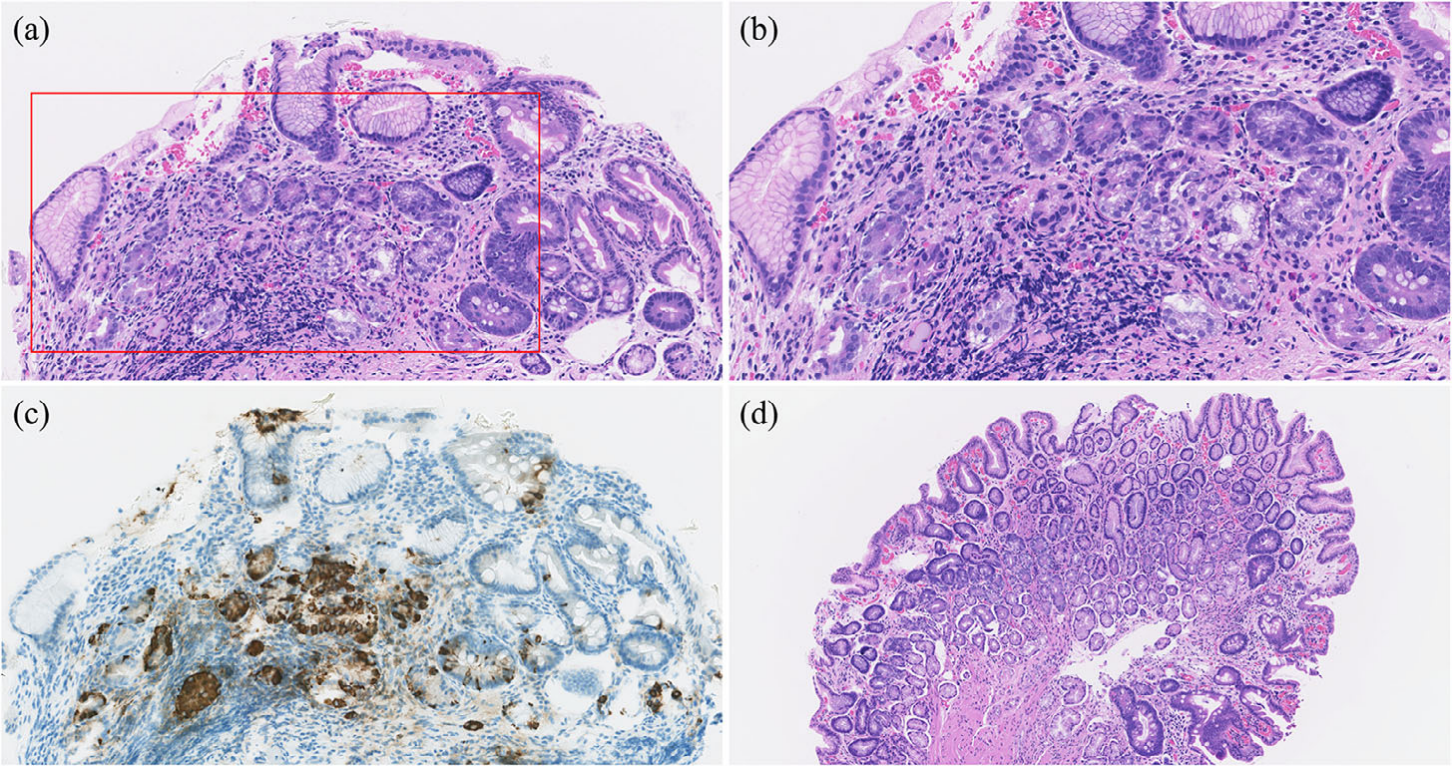
(a) A biopsy of the gastric body shows degeneration and loss of parietal cells; Decreased glandular density due to the progression of pseudopyloric gland metaplasia (mucous neck cell proliferation), foveolar elongation, and intense lymphocytic infiltration, mainly into tissues deeper than the isthmus, can be seen. Intestinal metaplasia is also observed (hematoxylin and eosin [HE] staining, × 40). (b) Magnified view of the area is indicated by the red frame in Figure 3a (HE staining, × 80). (c) Chromogranin A staining shows linear, tubular, and nodular hyperplasia of enterochromaffin‐like cells. (d) A biopsy of the greater curvature of the gastric antrum shows focal intestinal metaplasia. However, the degree of atrophy in the pyloric glands is less severe than in the oxyntic glands of the gastric body, with scarce lymphocytic infiltration. G‐cell hyperplasia is also observed (HE staining, × 40).

Pathological examination of biopsies from the gastric body (Figure 3a–c) and antrum (Figure 3d) revealed findings consistent with an advanced florid stage of AIG.^8^ Blood tests showed elevated APCA titers (160 ×; normal value <10×) and an increased serum gastrin level (425.9 pmol/L; normal range 11.9–46.9 pmol/L) in the absence of P‐CAB or H2RA. Furthermore, the pepsinogen I level and I/II ratio decreased. Endoscopic, histological, and serological findings confirmed the diagnosis of AIG.^6^ Furthermore, the histopathological findings from the second gastric body ESD resected specimen were consistent with those of an advanced florid stage of AIG complicated by *H. pylori* infection (Figure S1a,b).

## DISCUSSION

A multicenter study reported that PNS occurred in the distal stomach in 1.2% of post‐ESD cases.^7^ Excessive mucosal regeneration, peristaltic stimulation, bile reflux, *H. pylori* infection, gastric mucosal hyperplasia induced by H2RAs, proton pump inhibitors, and P‐CABs are possible mechanisms underlying PNS development.^2^, ^7^, ^10^ However, to our knowledge, no studies have investigated the relationship between serum gastrin levels and the development of PNS or a potential association between PNS and AIG.

As AIG progresses, severe atrophy of the gastric body occurs, leading to achlorhydria and hypergastrinemia through a negative feedback mechanism. Gastrin promotes gastric mucosal proliferation^8^ and stimulates gastric peristalsis.^9^ Hypergastrinemia‐induced excessive mucosal regeneration and increased peristaltic activity are consistent with the pathogenesis of PNS.^2^, ^7^ These findings suggest that excessive gastrin signaling contributes to the development of PNS by overstimulating mucosal repair mechanisms and enhancing gastric antral motility.

In this case, despite the eradication of *H. pylori* infection and adjustments to medications, the PNS continued to enlarge. Endoscopic and pathological examinations of the antrum after *H. pylori* eradication revealed minimal inflammation and bile acid reflux was of negligible impact. The involvement of hypergastrinemia was subsequently confirmed as causing excessive mucosal regeneration and increased peristalsis, and AIG was diagnosed through additional blood tests and pathological examinations.

In this case, ESD was performed at different anatomical locations, providing crucial insights into the mechanisms underlying PNS development. After *H. pylori* eradication, inflammation improved in the antrum but persisted in the gastric body owing to AIG. Under these conditions, the PNS continued to enlarge exclusively in the antral region but was not observed in the body, suggesting that PNS development is more strongly influenced by location‐specific factors in the antrum, such as strong peristalsis, than by background mucosal inflammation.

**FIGURE 4:**
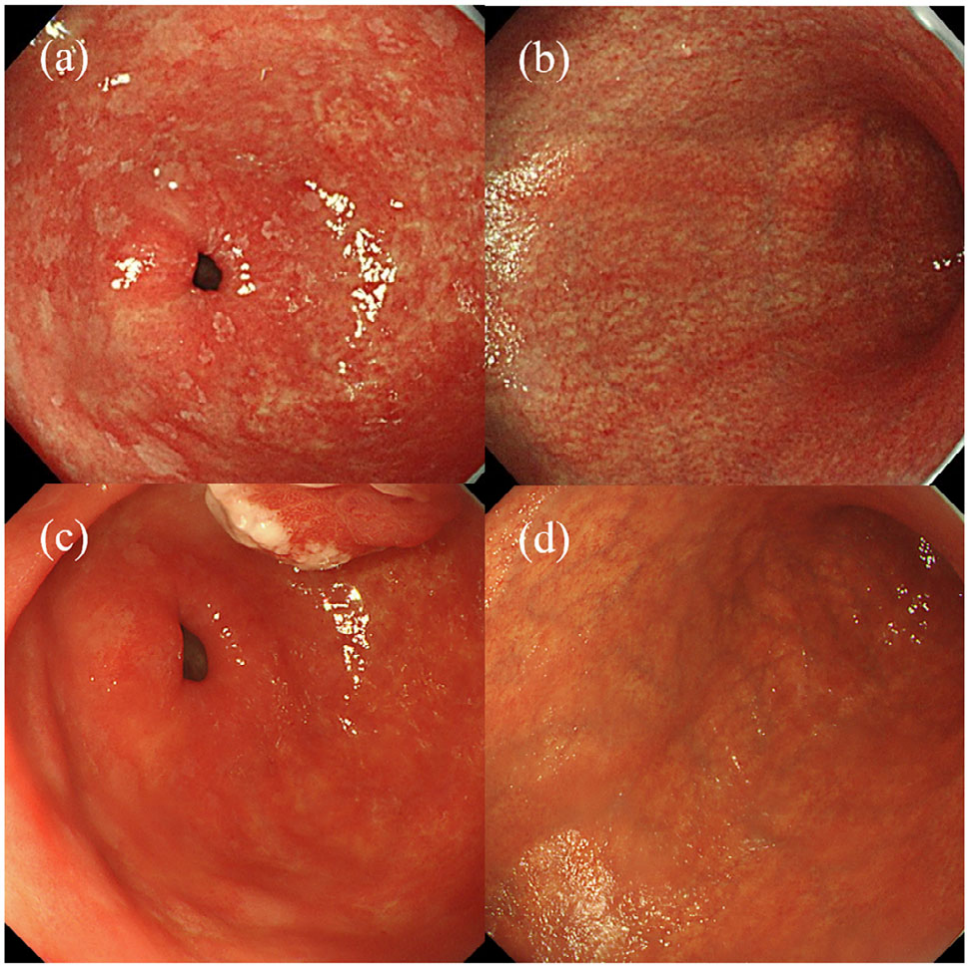
(a) Endoscopic images before *H. pylori* eradication show diffuse erythema and intestinal metaplasia in the gastric antrum which is associated with *H. pylori* infection. (b) Marked atrophy is also observed in the gastric body. (c) Endoscopic images after *H. pylori* eradication show that although intestinal metaplasia remains, no atrophic changes are observed in the gastric antrum because the inflammation has resolved, and the mucosa appears glossy. (d) In contrast, the gastric body exhibits marked atrophic changes.

Upon reviewing the pre‐ESD endoscopic images in this case, AIG was not initially suspected due to extensive intestinal metaplasia and mucosal erythema in the gastric antrum (Figure 4a), obscuring the characteristic “inverse atrophy” pattern of AIG. However, the gastric body exhibited extensive, marked atrophic changes, suggesting a possible concurrence of AIG and *H. pylori* infection (Figure 4b). Re‐evaluation of endoscopic imaging after *H. pylori* eradication showed glossy mucosa with residual intestinal metaplasia in the gastric antrum (Figure 4c) with persistent, significant gastric body atrophy (Figure 4d). Although *H. pylori* infection skewed gastric antrum imaging, pronounced gastric body atrophy compared to gastric antrum atrophy should have been considered characteristic of AIG.

PNS may recur after resection but may not require intervention in the absence of malignancy or symptomatic anemia. ESD combined with localized steroid injections has been proposed as a potential therapeutic option.^10^ Furthermore, PNS may be prevented by shortening the duration of proton pump inhibitors/PCABs or H2RA therapy and suturing the ulcer base to avoid exposure.

This case demonstrates that diagnosing the concurrence of active or previous *H. pylori* infection and AIG remains challenging. Clinicians should consider underlying hypergastrinemia in patients who develop PNS after ESD. AIG is an important differential diagnosis, potentially causing elevated gastrin levels. Although the association between PNS and AIG should be further studied, hypergastrinemia may be a clinically relevant risk factor for PNS in patients with AIG. To our knowledge, this is the first case report describing a potential link between AIG and PNS, offering a novel perspective for AIG diagnosis.

## CONFLICT OF INTEREST STATEMENT

None.

## ETHICS STATEMENT

Institutional Reviewer Board approval and Trial Registration are not applicable (N/A).

## PATIENT CONSENT STATEMENT

Informed consent was obtained from the patients.

## Supporting information




**FIGURE S1** (a) Reexamination of the resected specimen obtained from the second endoscopic submucosal dissection of the gastric body shows degeneration and loss of parietal and chief cells, pseudopyloric gland metaplasia (mucous neck cell proliferation), foveolar elongation, and intense lymphocytic infiltration, primarily in tissues deeper than the isthmus. Focal intestinal metaplasia is observed (hematoxylin and eosin staining, × 40). (b) Chromogranin A staining shows linear, tubular, and nodular hyperplasia of enterochromaffin‐like cells.

**FIGURE S2** The clinical course of the patient.
