# Supplementary figures and images for: Revealing autoimmune gastritis: Polypoid nodule scar development after endoscopic submucosal dissection for early gastric cancer
Source: DEN Open. 2025 Mar 11;5(1):e70094. doi: 10.1002/deo2.70094 (PMC11894263; doi:10.1002/deo2.70094)

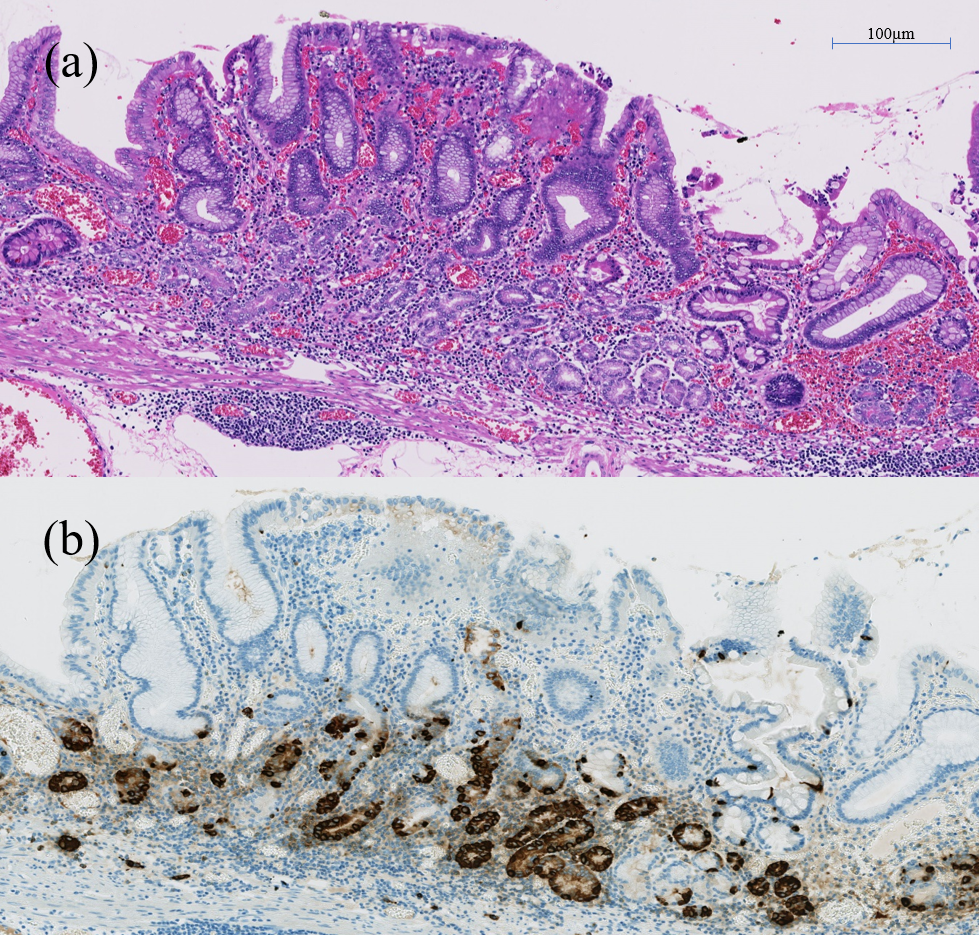

Supplement: Supplementary file 1 — FIGURE S1 (a) Reexamination of the resected specimen obtained from the second endoscopic submucosal dissection of the gastric body shows degeneration and loss of parietal and chief cells, pseudopyloric gland metaplasia (mucous neck cell proliferation), foveolar elongation, and intense lymphocytic infiltration, primarily in tissues deeper than the isthmus. Focal intestinal metaplasia is observed (hematoxylin and eosin staining, × 40). (b) Chromogranin A staining shows linear, tubular, and nodular hyperplasia of enterochromaffin‐like cells. [file DEO2-5-e70094-s001.tif]

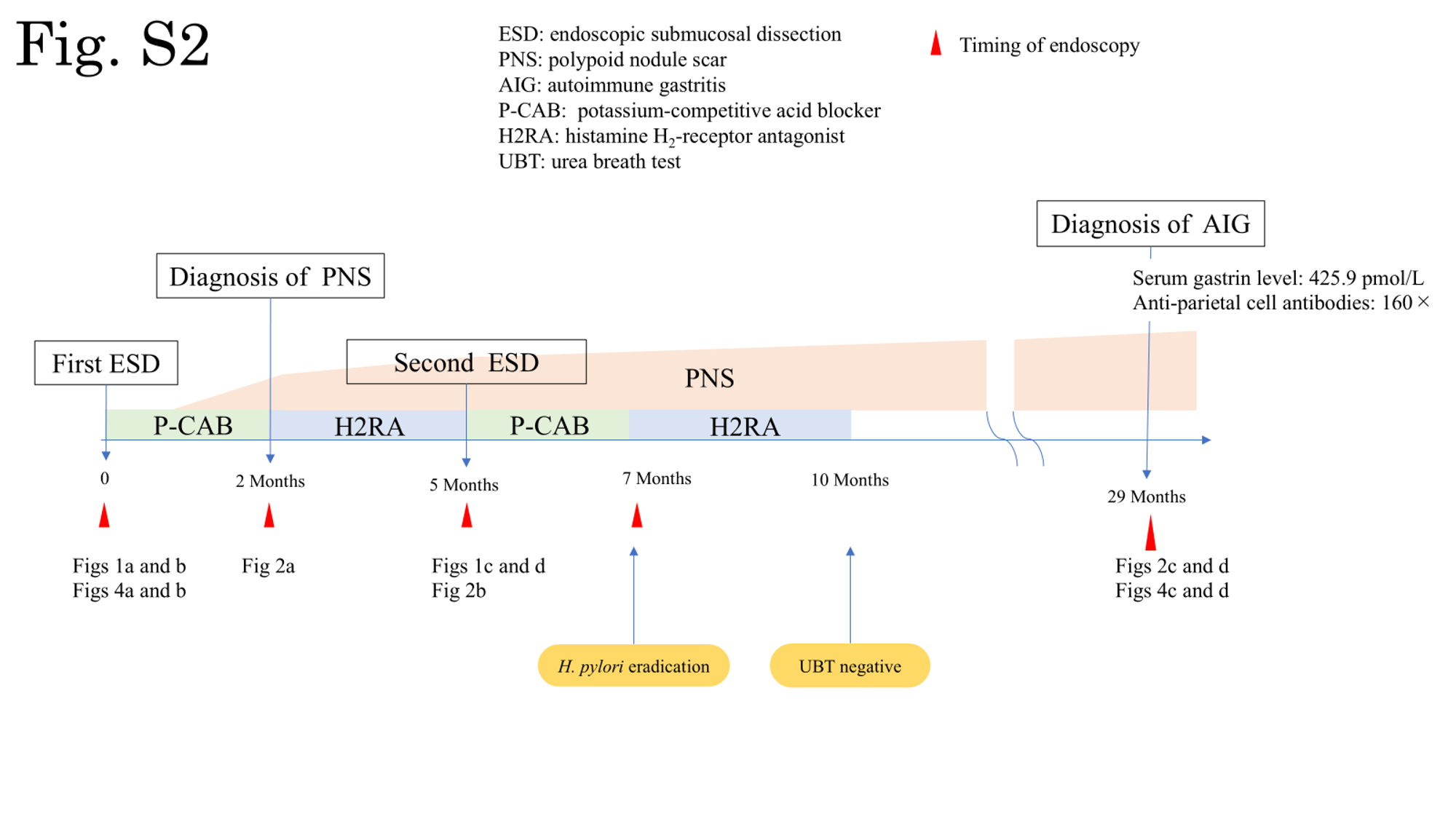

Supplement: Supplementary file 2 — FIGURE S2 The clinical course of the patient. [file DEO2-5-e70094-s002.tif]
